# Immune Thrombotic Thrombocytopenic Purpura Presenting as an Acute Stroke Mimic in a Young Pakistani Man

**DOI:** 10.7759/cureus.114729

**Published:** 2026-08-18

**Authors:** Muhammad Abbas, Zyad Saeed, Mahmoud Marashi, Deeb Kayed, Paddy Kilian, Abubakr Saaid

**Affiliations:** 1 Internal Medicine, Mediclinic City Hospital, Dubai, ARE; 2 Critical Care Medicine, Mediclinic City Hospital, Dubai, ARE; 3 Hematology, Mediclinic City Hospital, Dubai, ARE; 4 Neurology, Mediclinic City Hospital, Dubai, ARE; 5 Education, Mohammed Bin Rashid University of Medicine and Health Sciences (MBRU), Dubai, ARE; 6 Critical Care, Mediclinic City Hospital, Dubai, ARE

**Keywords:** immune thrombotic thrombocytopenic purpura, microangiopathic hemolytic anemia, plasma exchange, rituximab, stroke mimic

## Abstract

Immune thrombotic thrombocytopenic purpura (iTTP) is a life-threatening thrombotic microangiopathy that can have acute neurological symptoms and resemble ischemic stroke. A 27-year-old Pakistani man was transported by ambulance after being found unconscious, unable to speak, and unable to move his right side, which was suspected to be a stroke. He had been unwell for two weeks, was clinically jaundiced, and had a possible seizure during the previous week. Urgent plain computed tomography (CT) and CT angiography of the brain were called and revealed no acute abnormality and no obvious large vessel occlusion. There was no evidence of acute infarction on magnetic resonance imaging. His National Institutes of Health Stroke Scale (NIHSS) score was 15/42, which is a moderate stroke. Laboratory studies showed hemoglobin 6.0 g/dL, platelet count 3x10^9^/L, schistocytes on peripheral smear, lactate dehydrogenase 2139 U/L, haptoglobin <0.01 g/L, reticulocytosis, and mainly indirect hyperbilirubinemia. The clinical features were compatible with iTTP complicated with neurological involvement. The patient was treated with therapeutic plasma exchange daily, high-dose methylprednisolone, and rituximab. Platelets rose steadily to 199x10^9^/L on day 8, bilirubin levels dropped to 14.2 µmol/L, and lactate dehydrogenase levels dropped to 259 U/L at discharge. Neurological recovery was quick, with resolution of the right-sided weakness by the fourth hospital day and an NIHSS score of 0. This case highlights that in young patients with stroke-like presentation, severe thrombocytopenia, and microangiopathic hemolytic anemia, prompt plasma exchange and immunosuppression can be lifesaving and should be considered in iTTP.

## Introduction

Thrombotic thrombocytopenic purpura (TTP) is a rare but potentially fatal thrombotic microangiopathy characterized by microangiopathic hemolytic anemia, thrombocytopenia, and variable organ ischemia. TTP results from severe deficiency of a disintegrin and metalloproteinase with thrombospondin type 1 motif, member 13 (ADAMTS13). In acquired immune-mediated TTP, anti-ADAMTS13 autoantibodies cause severe ADAMTS13 deficiency, whereas congenital TTP results from pathogenic variants in the ADAMTS13 gene [1-3].

The classic pentad of fever, neurological dysfunction, renal impairment, hemolytic anemia, and thrombocytopenia is neither required for diagnosis nor commonly complete at presentation [1,2]. Severe thrombocytopenia with microangiopathic hemolytic anemia should therefore prompt urgent consideration of TTP, particularly when neurological abnormalities are present. ADAMTS13 testing is essential for confirmation, but treatment should not be delayed when the clinical probability is high [1,2].

Neurological involvement may include confusion, seizures, coma, transient focal deficits, or stroke-like hemiparesis. Such presentations can activate an acute stroke pathway, especially in a young adult with sudden focal weakness, yet concomitant thrombocytopenia and microangiopathic hemolysis should prompt consideration of TTP. We describe severe ADAMTS13-deficient TTP presenting as an acute stroke mimic with normal acute neuroimaging and a possible seizure-related postictal deficit. The specific educational contribution is the diagnostic uncertainty created by the coexistence of focal neurological findings, a possible seizure, profound hematological abnormalities, and no corresponding acute ischemic lesion, requiring integration of neurological, hematological, and imaging evidence rather than attribution to a single mechanism.

## Case presentation

A 27-year-old Pakistani man was brought to the emergency department by the Dubai ambulance service with suspected acute stroke on March 30, 2026 (hospital day 0). He had gone to bed the previous night without a major neurological complaint. He had been generally unwell for approximately two weeks but had not received treatment because of lack of insurance coverage. He was jaundiced at presentation. There was also a history suggestive of a seizure during the preceding week, although details of that event were unavailable; given that he was found altered after sleep, an unwitnessed nocturnal seizure at presentation could not be excluded.

**Figure 1:**
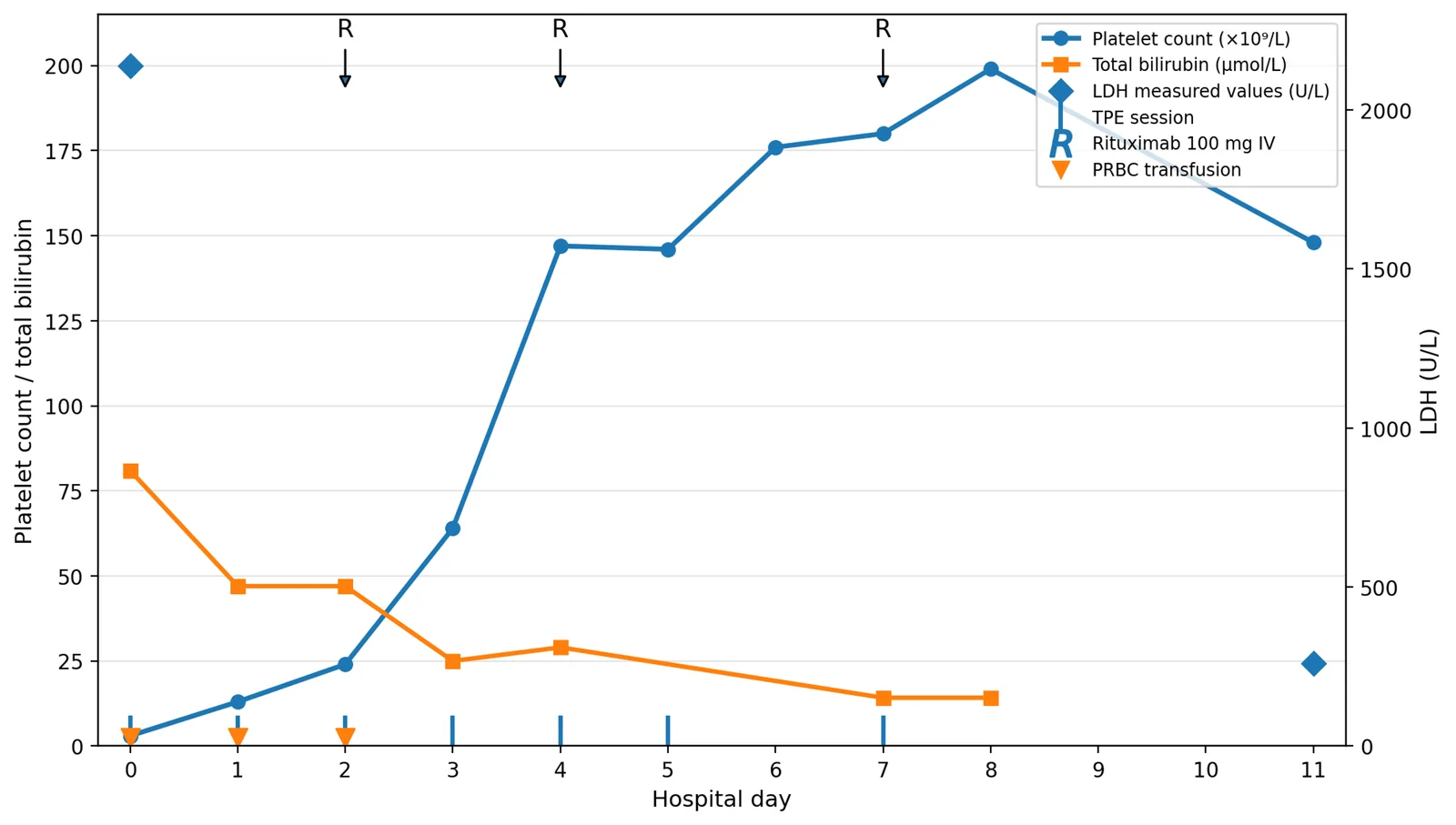
Trends in platelet count, total bilirubin, and measured LDH values during admission Short vertical marks on the x-axis show individual therapeutic plasma exchange sessions. “R” marks rituximab 100 mg IV administered on hospital days 2, 4, and 7, while downward triangles indicate packed red blood cell transfusions on hospital days 0, 1, and 2. LDH is shown only at admission and hospital day 11 because intermediate values were unavailable. IV, intravenous; LDH, lactate dehydrogenase; PRBC, packed red blood cell; TPE, therapeutic plasma exchange.

On the morning of presentation, his flatmate attempted to wake him and found him with altered consciousness, aphasia, and right hemiparesis. Code stroke was activated, and his National Institutes of Health Stroke Scale (NIHSS) score was 15 [4]. Computed tomography (CT) of the brain showed no acute abnormality. CT angiography showed no cerebral large-vessel occlusion, and magnetic resonance imaging (MRI) performed with intravenous contrast included diffusion-weighted imaging (DWI), apparent diffusion coefficient maps, T2-weighted imaging, fluid-attenuated inversion recovery, susceptibility-weighted imaging, post-contrast sequences, and three-dimensional time-of-flight magnetic resonance angiography. DWI showed no restricted diffusion to indicate an acute infarction, and magnetic resonance angiography showed no major intracranial vessel occlusion. Representative admission neuroimaging is shown in Figure 1.

**Figure 2:**
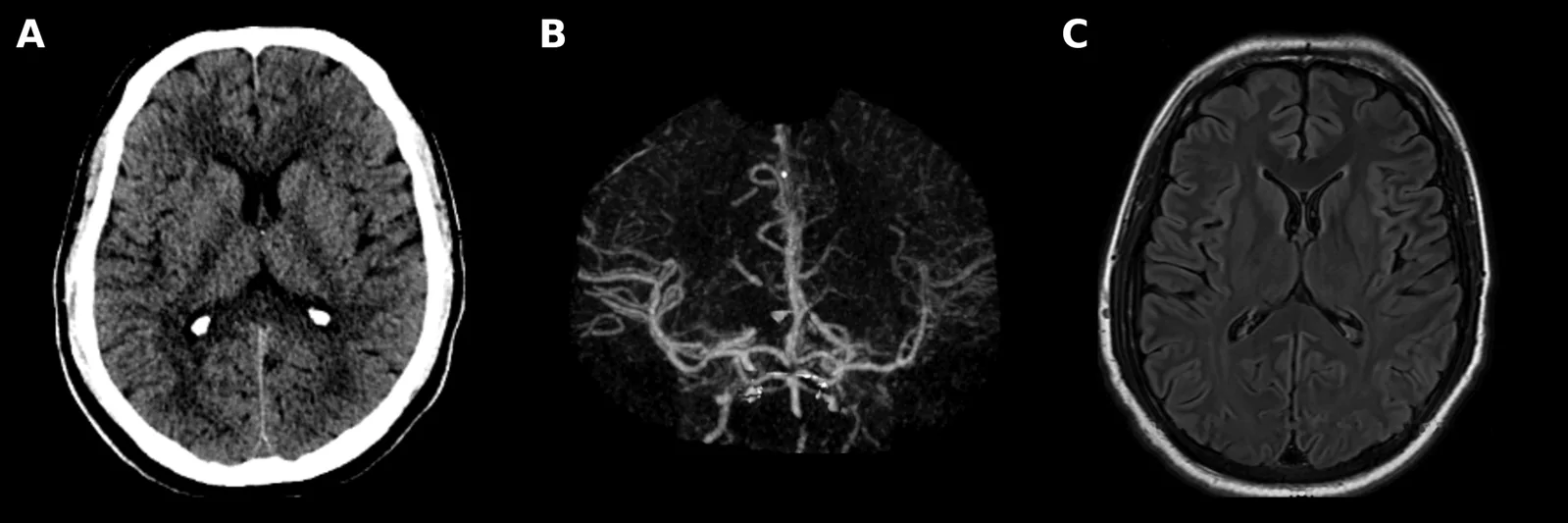
Representative admission neuroimaging (A) Axial non-contrast CT brain showing no acute intracranial abnormality. (B) CT angiography maximum-intensity projection showing no cerebral large-vessel occlusion. (C) Axial fluid-attenuated inversion recovery MRI showing no focal acute parenchymal abnormality. CT, computed tomography; MRI, magnetic resonance imaging.

Initial neuroimaging included non-contrast CT of the brain, which showed no acute abnormality; CT angiography, which showed no cerebral large vessel occlusion; and MRI, which showed no acute infarction or major vessel occlusion. Representative admission neuroimaging is shown in Figure 2.

At hematology assessment, the patient was unarousable after receiving benzodiazepine sedation for MRI. The sedative effect may have contributed to or prolonged the altered level of consciousness and limited the reliability of neurological assessment during this period; it could not, however, account for the profound hematological abnormalities. Right-sided weakness persisted. There was no organomegaly; vital signs were stable, and oxygenation was maintained on room air. The first blood results were striking: hemoglobin was 6.0 g/dL and the platelet count only 3×10⁹/L. The blood film showed numerous schistocytes, with polychromasia and nucleated red cells, in keeping with microangiopathic hemolysis. Lactate dehydrogenase (LDH) was 2139 U/L, haptoglobin <0.01 g/L, the absolute reticulocyte count 414×10⁹/L, and total bilirubin 81 µmol/L, mainly indirect. The prothrombin time was mildly prolonged, while the activated partial thromboplastin time remained normal.

Taken together, the severe thrombocytopenia, hemolytic blood film, biochemical evidence of hemolysis, and neurological findings made TTP the leading diagnosis. A formal PLASMIC score or another validated clinical prediction score was not prospectively documented. Blood for ADAMTS13 activity and anti-ADAMTS13 antibody testing was obtained before therapeutic plasma exchange; ADAMTS13 activity was not repeated after plasma exchange. ADAMTS13 activity was subsequently reported as 7.1% (reference value >66.8%), confirming severe ADAMTS13 deficiency. The anti-ADAMTS13 antibody titer was 11 (reference <12), immediately below the assay cutoff. Given the adult-onset presentation, absence of a history suggestive of congenital TTP, and clinical response to immunosuppression, an acquired immune-mediated mechanism was considered most likely; however, the borderline-negative antibody result precluded definitive serological classification, and the immune-mediated etiology therefore remained presumed [1-3]. An autoimmune evaluation, including assessment for systemic lupus erythematosus and antiphospholipid syndrome, was performed and did not identify an abnormality.

The case was discussed promptly with the critical care and hematology teams. Intravenous thrombolysis and antiplatelet therapy were withheld because the platelet count was 3×10⁹/L and the associated bleeding risk was considered prohibitive. Intravenous methylprednisolone 500 mg/day was initiated with proton pump inhibitor cover, and urgent therapeutic plasma exchange was started using a 4-L exchange volume per session without waiting for the ADAMTS13 result. Rituximab 100 mg was administered intravenously after plasma exchange on April 1 (hospital day 2), April 3 (hospital day 4), and April 6, 2026 (hospital day 7). This low fixed-dose, three-dose schedule was an individualized decision by the treating hematology team and should not be interpreted as a standard rituximab regimen for TTP. Methylprednisolone was reduced to 80 mg/day on hospital day 4. For severe symptomatic anemia, the patient received one unit of packed red blood cells on each of hospital days 0, 1, and 2. No platelet transfusion was administered. Caplacizumab was not administered; the available clinical records did not document a specific reason for its omission.

On hospital day 1, the patient remained unarousable with persistent right-sided hemiplegia and developed fever up to 39°C. Antimicrobial therapy was reviewed and optimized by the intensive care team. By hospital day 2, he responded to voice, obeyed commands, and moved the right leg, although the right arm remained weak. The following day he was fully awake, answered simple questions appropriately, and moved all four limbs without a clear residual focal deficit. His subsequent NIHSS score was 0. The platelet count had increased to 64×10⁹/L, total bilirubin decreased to 25 µmol/L, and creatinine improved from a peak of 126 µmol/L to 86 µmol/L.

Neurological recovery continued. He was transiently confused on hospital day 4 but was mentally clear by hospital day 5. The platelet count increased to 147×10⁹/L on hospital day 4, 176×10⁹/L on hospital day 6, 180×10⁹/L on hospital day 7, and 199×10⁹/L on hospital day 8. The central venous catheter was removed after the final plasma exchange and rituximab infusion. At repatriation on April 10, 2026 (hospital day 11), he was clinically well, with hemoglobin 11.0 g/dL, platelet count 148×10⁹/L, and LDH 259 U/L. At repatriation, he was receiving a tapering course of prednisolone and was advised to receive one further rituximab 100 mg intravenous infusion one week later in his home country. The clinical course and treatment are summarized in Table 1, while the principal laboratory changes are presented in Table 2 and Figure 2.

**Table 1:** Clinical course, treatment, and outcome CTA, computed tomography angiography; CT, computed tomography; IV, intravenous; LDH, lactate dehydrogenase; MRI, magnetic resonance imaging; NIHSS, National Institutes of Health Stroke Scale; PRBC, packed red blood cell; TTP, thrombotic thrombocytopenic purpura.

Hospital day	Clinical and laboratory status	Management and outcome
Laboratory units/reference ranges	Hemoglobin, g/dL (13.0-17.0); platelet count, ×10⁹/L (150-400); total bilirubin, µmol/L (3-21); creatinine, µmol/L (62-106); LDH, U/L (135-225); haptoglobin, g/L (reference interval unavailable)	
Hospital day 0	Confusion, mutism, right-sided weakness, and jaundice; NIHSS 15; CT showed no acute abnormality; CTA showed no large vessel occlusion; MRI showed no acute infarction; hemoglobin 6.0 g/dL; platelets 3×10⁹/L; schistocytes; LDH 2139 U/L; haptoglobin <0.01 g/L; bilirubin 81 µmol/L	TTP suspected; methylprednisolone 250 mg/day and urgent 4-L plasma exchange initiated; one unit PRBC transfused
Hospital day 1	Persistent unresponsiveness and right hemiplegia; fever 39°C; platelets 13×10⁹/L; bilirubin 47 µmol/L; creatinine 126 µmol/L	Plasma exchange and high-dose methylprednisolone continued; antimicrobial therapy optimized; one unit PRBC transfused
Hospital day 2	Responded to voice and followed commands; right leg movement returned, but right upper limb weakness persisted; platelets 24×10⁹/L	Plasma exchange continued; rituximab 100 mg IV administered after exchange; one unit PRBC transfused
Hospital day 3	Fully conscious; moved all limbs without obvious focal weakness; subsequent NIHSS 0; platelets 64×10⁹/L; bilirubin 25 µmol/L; creatinine 86 µmol/L	Plasma exchange and corticosteroids continued
Hospital day 4	Fully conscious but transiently confused; platelets 147×10⁹/L	Plasma exchange continued; methylprednisolone reduced to 80 mg/day; rituximab 100 mg IV repeated
Hospital days 5-8	Mental status normalized; platelet count increased from 146 to 199×10⁹/L; hemoglobin reached 11.0 g/dL	Plasma exchange course completed, including final session and rituximab on hospital day 7; central venous catheter removed
Hospital day 11	Clinically well at repatriation; hemoglobin 11.0 g/dL; platelets 148×10⁹/L; LDH 259 U/L	Prednisolone taper continued; one additional rituximab dose advised in home country

**Table 2:** Key laboratory trends during admission Reference intervals: hemoglobin, 13.0-17.0 g/dL; platelet count, 150-400×10⁹/L; total bilirubin, 3-21 µmol/L; creatinine, 62-106 µmol/L; LDH, 135-225 U/L; and CRP, <5 mg/L. Three units of packed red blood cells were administered, one each on hospital days 0, 1, and 2. Intermediate CRP measurements were 44, 16, and 6.2 mg/L; their corresponding hospital days were unavailable. CRP, C-reactive protein; LDH, lactate dehydrogenase.

Hospital day	Hemoglobin (g/dL)	Platelet count (×10⁹/L)	Total bilirubin (µmol/L)	Creatinine (µmol/L)	LDH (U/L)	CRP (mg/L)
Hospital day 0	6	3	81	Not available	2139	Not available
Hospital day 1	Not available	13	47	126	Not available	Not available
Hospital day 2	Not available	24	47	Not available	Not available	Not available
Hospital day 3	Not available	64	25	86	Not available	Not available
Hospital day 4	Not available	147	Not available	86	Not available	Not available
Hospital day 5	Not available	146	Not available	Not available	Not available	Not available
Hospital day 6	Not available	176	Not available	Not available	Not available	Not available
Hospital day 7	Not available	180	14.2	Not available	Not available	Not available
Hospital day 8	11	199	14.2	81	Not available	3.2
Hospital day 11	11	148	Not available	Not available	259	Not available

## Discussion

At presentation, the neurological picture was convincing enough to activate the stroke pathway: sudden mutism, confusion, right-sided weakness, and a possible recent seizure. Yet CT, CT angiography, and MRI did not show an acute infarct or large-vessel occlusion. This combination created a clinically important stroke-mimic scenario. The profound thrombocytopenia, blood-film evidence of microangiopathic hemolysis, and subsequently confirmed severe ADAMTS13 deficiency made TTP the leading systemic diagnosis. These findings established the need to treat TTP urgently but did not establish the mechanism of the transient neurological deficit. Because the patient was found altered after sleep and had a possible preceding seizure-like event, a postictal state with Todd's paralysis remained a plausible alternative explanation for the transient hemiparesis. The benzodiazepine administered for MRI may also have contributed to the depressed consciousness and confounded early neurological assessment.

Neurological involvement in TTP varies considerably and can change over a matter of hours. Some patients have headache or confusion, whereas others develop seizures, coma, transient focal deficits, or ischemic stroke [3]. Proposed mechanisms include microvascular ischemia, but normal acute neuroimaging in this patient provides no direct evidence of cerebral microthrombosis and does not establish the mechanism of the focal deficit. The neurological presentation could have reflected TTP-associated dysfunction, a seizure with postictal paresis, or a combination of factors. This uncertainty is central to the educational value of the case.

This distinction from ordinary acute ischemic stroke matters at the bedside. In this patient, intravenous thrombolysis and antiplatelet therapy were withheld because profound thrombocytopenia created an unacceptable bleeding risk. Suspected TTP, in contrast, requires urgent disease-specific treatment. Guidelines advise obtaining an ADAMTS13 sample as early as possible, but treatment should begin on clinical grounds when the probability of TTP is high [1,2]. In this case, the sample was collected before plasma exchange, although a formal PLASMIC score was not prospectively documented. Plasma exchange remains the mainstay of acute therapy because its survival benefit was demonstrated in a randomized trial [5]. Corticosteroids are used for immunosuppression, and rituximab is commonly added in immune TTP. Both conventional and low fixed-dose rituximab regimens have been reported [2,6,7], but the 100-mg schedule on hospital days 2, 4, and 7, in this case, was individualized by the treating hematology team and is not an established standard regimen.

Treatment was started without waiting for the ADAMTS13 result because the clinical and laboratory findings were highly suggestive of TTP. Severe ADAMTS13 deficiency subsequently supported the diagnosis. Platelets increased from 3 to 199×10⁹/L, bilirubin decreased from 81 to 14.2 µmol/L, LDH decreased from 2139 to 259 U/L, and the focal neurological deficit resolved over the following days. These improvements occurred temporally after initiation of plasma exchange, corticosteroids, and rituximab; however, this single case cannot establish that TTP-directed therapy directly caused the neurological recovery, particularly because postictal paresis remained possible. Other reports have similarly described initiating treatment from the clinical presentation and routine laboratory findings while awaiting ADAMTS13 testing [8,9].

Caplacizumab was not administered, and the available records did not document a specific reason; its omission is therefore reported without attributing a retrospective rationale. Caplacizumab blocks the interaction between von Willebrand factor and platelets and, in a randomized trial, shortened the time to platelet-count normalization [10]. The 2025 focused update of the International Society on Thrombosis and Haemostasis (ISTH) treatment guidelines reviewed additional evidence supporting caplacizumab in combination with plasma exchange and immunosuppressive therapy and retained the 2020 recommendations for immune TTP [11]. A recent review summarizes its role in contemporary immune TTP management, including reduced exacerbations during treatment and an increased risk of bleeding [12]. Real-world outcome data also support early caplacizumab use as part of multimodal treatment for immune TTP [13]. Considerations such as access, cost, bleeding risk, and local protocols can influence its use generally, but these factors cannot be assumed to explain its omission in this patient.

This case has several limitations. The anti-ADAMTS13 antibody result was immediately below the laboratory cutoff; consequently, the immune-mediated etiology was presumed from the adult-onset clinical context, absence of features suggesting congenital TTP, severe ADAMTS13 deficiency, and response to immunosuppression, rather than definitively established serologically. The mechanism of the neurological deficit also remains uncertain: acute imaging showed no ischemic lesion, an unwitnessed nocturnal seizure with postictal Todd's paralysis was possible, and benzodiazepine sedation confounded assessment of consciousness. A formal PLASMIC score was not prospectively documented, and the reason caplacizumab was not administered was unavailable in the records. Information regarding the earlier possible seizure, source of infection, and full antimicrobial treatment was incomplete. ADAMTS13 activity was not repeated after plasma exchange, and there were no intermediate LDH measurements. Finally, the patient was lost to follow-up after repatriation to his home country; therefore, long-term neurological and hematological follow-up was unavailable, preventing assessment of sustained neurological recovery, durable hematological remission, subsequent ADAMTS13 activity, and relapse.

## Conclusions

This case highlights the importance of considering TTP in the differential diagnosis of a young patient with acute focal neurological deficits accompanied by severe thrombocytopenia and microangiopathic hemolytic anemia. Normal acute neuroimaging did not establish the mechanism of the neurological deficit, and a preceding seizure with postictal Todd's paralysis remained a plausible alternative explanation. The observed neurological and hematological improvement occurred after initiation of plasma exchange, corticosteroids, and rituximab, but a definitive causal relationship cannot be inferred from a single case. The findings should therefore be interpreted cautiously and alongside established diagnostic and treatment guidelines.
